# Stressful environments induce novel phenotypic variation: hierarchical reaction norms for sperm performance of a pervasive invader

**DOI:** 10.1002/ece3.364

**Published:** 2012-09-13

**Authors:** Craig F Purchase, Darek T R Moreau

**Affiliations:** Fish Evolutionary Ecology Research Group, Departments of Biology & Ocean Sciences, Memorial UniversitySt. John's, Newfoundland and Labrador, Canada

**Keywords:** Brown trout, CASA, cryptic variation, fertilization, genotype × environment interaction, invasive species, pH, phenotypic plasticity, *Salmo trutta*, sperm quality

## Abstract

Genetic variation for phenotypic plasticity is ubiquitous and important. However, the scale of such variation including the relative variability present in reaction norms among different hierarchies of biological organization (e.g., individuals, populations, and closely related species) is unknown. Complicating interpretation is a trade-off in environmental scale. As plasticity can only be inferred over the range of environments tested, experiments focusing on fine tuned responses to normal or benign conditions may miss cryptic phenotypic variation expressed under novel or stressful environments. Here, we sought to discern the presence and shape of plasticity in the performance of brown trout sperm as a function of optimal to extremely stressful river pH, and demarcate if the reaction norm varies among genotypes. Our overarching goal was to determine if deteriorating environmental quality increases expressed variation among individuals. A more applied aim was to ascertain whether maintaining sperm performance over a wide pH range could help explain how brown trout are able to invade diverse river systems when transplanted outside of their native range. Individuals differed in their reaction norms of phenotypic expression of an important trait in response to environmental change. Cryptic variation was revealed under stressful conditions, evidenced through increasing among-individual variability. Importantly, data on population averages masked this variability in plasticity. In addition, canalized reaction norms in sperm swimming velocities of many individuals over a very large range in water chemistry may help explain why brown trout are able to colonize a wide variety of habitats.

## Introduction

Phenotypes vary due to genetic differences in plasticity and genetic differences that impart common expression across environments. For instance, species differences in size, shape, or physiological function of a mouse and a penguin are present under any condition. Phenotypic plasticity, however, occurs when a single genotype is expressed as multiple phenotypes when exposed to environmental variation (Schlichting and Pigliucci 1998; West-Eberhard 2003). The profile of this plasticity is referred to as a reaction norm (Woltereck 1909; Schlichting and Pigliucci 1998), and this may vary among genotypes. Although difficult to determine, plasticity has important ecological and evolutionary consequences. For example, depending on the context, plasticity can speed up or slow down the rate of adaptation to a given selective pressure (Price et al. 2003; Ghalambor et al. 2007).

Plasticity may or may not be adaptive depending on the trait and situation. It is adaptive when plasticity produces a better phenotype/environment match over a wider range of environments than would otherwise occur, or in other words, if it allows a specific genotype to endure more environmental variability. If a constant phenotype is optimal, then a flat or “canalized” reaction norm is adaptive and the presence of plasticity is maladaptive (Eshel and Matessi 1998). Having the developmental mechanisms to exhibit plasticity can be thought of as a trait in itself. Like others, the ability to produce this trait (plasticity) carries costs, which may maintain genetic variation for it (see DeWitt et al. 1998). Not surprisingly, both the intercept and slope of a reaction norm can be under selection (Gavrilets and Scheiner 1993), and vary among genotypes.

As selection acts on individuals, it is at this hierarchical level of biological organization where the greatest insights on plasticity are likely to be made. Individual or within-genotype plasticity is hard to measure, especially in natural populations (Nussey et al. 2007) and, as a result, much empirical work focuses on environmental comparisons across siblings or populations. Importantly, although population-level reaction norms depend on individuals, individual patterns cannot explicitly be inferred from population averages (Nussey et al. 2007). When individual reaction norms are available, experimental designs have typically been quite simple, such as monitoring changes in reproduction of birds or mammals in different years.

A novel and powerful tool for studies of individual plasticity are the sperm of external fertilizers (Purchase et al. 2010), such as most fishes. Complex experiments are possible because sperm cells from the same ejaculate can be examined under different environments. Although individual sperm transport unique haploid genetic material, research has shown that sperm morphology and behavior is under dipoid control of the father (see references within Purchase et al. 2010), and thus performance of all sperm from an ejaculate is related to the single paternal genotype. West-Eberhard (2003) wrote eloquently “Although a complex, motile sperm cell looks like an independent little individual organism (fig. 5.7 *in her book*), the genes of the animal spermatozoan in most species are physically condensed and completely inert (Baccetti and Afzelius 1976) during the gamete stage. The morphological, biochemical, and behavioral phenotype of the spermatozoan is a product of the paternal phenotype, not the genes within the sperm”. As single cells, sperm are very sensitive to environmental variability and external fertilizers, such as most fishes release sperm into environments that can vary in temperature and/or chemistry. In addition, most fish sperm have very short lifespans, allowing plasticity to be assessed easily for swimming performance, which is tightly linked to fitness. If males are sampled from the wild, their past experiences may impart a non-genetic effect on the performance of their sperm and reaction norms of individuals would not necessarily reflect the reaction norm of the specific genotype (see general arguments in Nussey et al. 2007). If males, however, have been reared under common conditions, then sperm reaction norms of individuals should approach equivalence to those of specific genotypes (Purchase et al. 2010). Here, we employ such an approach to questions related to the exposure of an invasive species to stressful environments.

**Figure 1:**
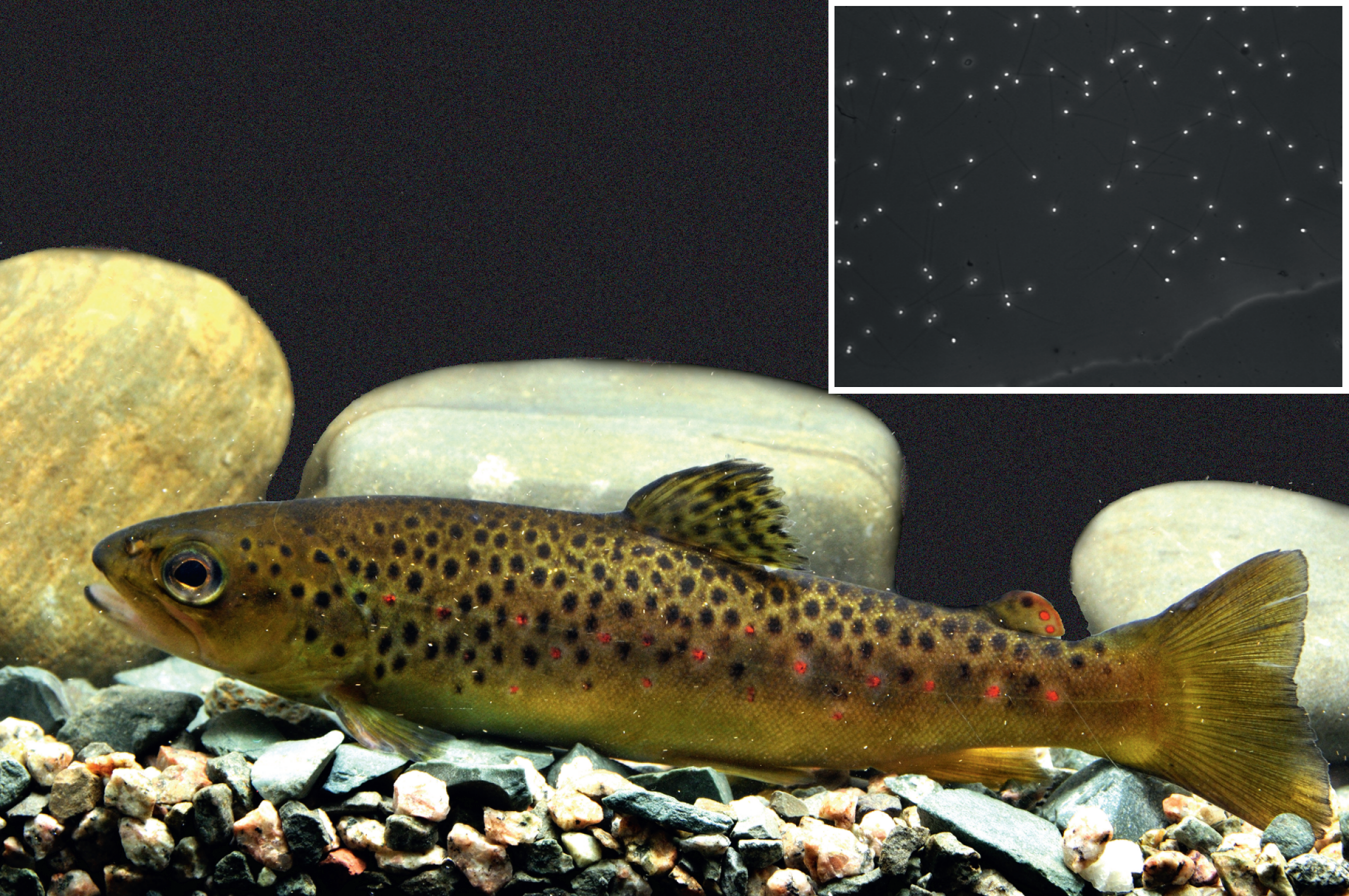
A young brown trout (*Salmo trutta*), photo credit Peter Westley. Insert: heads of brown trout sperm appear as bright white dots under phase contrast at 400×, photo credit Craig Purchase.

Several authors have recently investigated the role of phenotypic plasticity in studying species invasions (Richards et al. 2006; Davidson et al. 2011; Westley 2011). Increasingly, the establishment of non-native species is precipitated by unintentional or deliberate anthropogenic activity. Purposeful introductions of salmonid fish have made them one of the most uprooted taxa on the planet. In recent years, several non-native species and populations have been escaping from aquaculture operations and entering adjacent river systems. However, salmonid introductions have a long history through recreational angling. For example, when British colonists travelled the world they often brought plants and animals that reminded them of home. Among these species was the brown trout (*Salmo trutta* Linnaeus, Fig. 1), which is native to Eurasia and has become well established in many temperate regions of the globe. They are of conservation concern to native biodiversity in many areas (see references within Westley and Fleming 2011) and have been labeled one of the “100 worst invasive alien species” of any taxa (Lowe et al. 2000).

Britain's oldest former colony is Newfoundland, a large temperate island off the east coast of North America. Brown trout embryos were shipped to the capital city of St. John's from Scotland from 1883 to 1906 and stocked into 16 watersheds (Hustins 2007). Surviving trout had access to the sea as early as 1884 and have since established populations in at least 51 new systems (Westley and Fleming 2011). The watersheds in eastern Newfoundland vary somewhat in chemistry and those of higher conductivity seem more likely to be invaded (Westley and Fleming 2011). As brown trout continue to move around the coast of Newfoundland, they will encounter more variable rivers, including some that drain from exposed granite bedrock and others from limestone or serpentine barrens (known pH range of a small subset of rivers 4.7–7.8, *N* = 111; http://www.public.geoportal-geoportail.gc.ca/dfoGeoPortal/).

Water chemistry influences reproductive success in salmonids; many populations have been eliminated due to acid rain. For brown trout, the reproductive rate at pH 5.5 is only 50% of that at pH 6.0 and Atlantic salmon (*Salmo salar* Linnaeus), a congener, are negatively affected even at pH 6.3 (see Jonsson and Jonsson 2011). Intense research on embryos, alevins, and juveniles has shown that reproduction completely fails in the genus *Salmo* at pH 4 (Daye and Garside 1977; Peterson et al. 1980; Serrano et al. 2008). Separating potential causal mechanisms is difficult and how stressful pH levels affect fertilization capacity of sperm in the wild is unknown. In artificial aquaculture settings, maximum fertilization rates are desirable and optimal results are achieved under alkaline environments (see Alavi and Cosson 2005).

Here, we sought to discern the presence and shape of plasticity in the performance of brown trout sperm as a function of optimal to extremely stressful river pH, and demarcate if the reaction norm varies among genotypes. Our overarching goal was to determine if deteriorating environmental quality increases expressed variation among individuals in a trait tightly linked to fitness. A more applied aim was to ascertain whether maintaining sperm performance over a wide pH range could help explain how brown trout are able to colonize so many diverse river systems.

## Materials and Methods

### Fish history

The wild-sourced parents came from two streams in St. John's, Newfoundland, Canada, which were among the first documented sites of the introduction of brown trout to the island in the 1880s (Hustins 2007). Middle Rocky Pond Brook (general conductivity 44 ± 0.5 μS/m, pH 5.8 ± 0.20) flows through forest into Windsor Lake, a major water supply for the city. Rennie's River (general conductivity 246 ± 17.1 μS/m, pH 7.0 ± 0.09) runs through the middle of the city and receives anthropogenic inputs. These watersheds contain reproductively isolated brown trout populations and were chosen due to convenience and not because of substantial environmental or genetic variability between them. Adult fish were collected in autumn of 2008 and gametes were stripped and used to create eight full families from each population. These families were laboratory-reared in adjacent tanks under common ambient conditions until mature; separately through embryo incubation, and then pooled by population before being tagged at 8 months of age and subsequently combined into a single tank. Unfortunately, pedigree information is not available.

### Sperm collection, preparation, and assessment

In November 2010, 20 mature F_1_ captive males from Middle Rocky Pond Brook and Rennie's River sourced parents were assessed for sperm performance under different conditions. Mean fish length was similar between populations [Rennie's River = 204 ± 12.5 mm SD, Middle Rocky Pond Brook = 211 ± 21.2 mm; Table 1]. Fish were lightly anesthetized with MS-222 (Western Chemical Inc., Ferndale, NJ) and semen was collected in 1-mL plastic syringes before being stored in 1.5-mL centrifuge tubes. Any samples obviously contaminated with feces or urine were discarded. As in most species of fish, brown trout sperm are inactive inside the male, but start swimming immediately on contact with water of appropriate chemistry. Semen samples were pre-diluted in a non-activating medium to enable simultaneous activation of all sperm on a slide, and consistent mixing for microscope images. Approximately, 0.5 mL of semen was centrifuged at 5°C for 10 min at 4100 g to obtain seminal plasma. Uncentrifuged semen samples were then diluted 1:100 by adding centrifuged plasma from the same fish, a process that did not activate the sperm. All sperm analyses were conducted at 5°C, the ambient temperature at the time semen was sampled. For each procedure, a diluted semen aliquot of 5 μL was placed into a 8-mm well of a temperature controlled multi-test slide, followed quickly by 40 μL of 5°C activating water solution (see below). This method enabled quick and consistent mixing of semen with test water.

**Table 1:** Summary data for individual males from Middle Rocky Pond Brook (M) and Rennie's River (R) sourced parents. Mean velocities shown for each fish are 10-sec post-activation and are averages among five pH treatments, among five procedural replicates, among cells on a slide. Figure 5 shows individual reaction norms of VCL at 10-sec post-activation; here the pH of peak velocity is indicated

Stream	Fish	Date assessed	Fork length (mm)	Mean VCL (μm/sec)	Mean VAP (μm/sec)	Mean VSL (μm/sec)	pH peak velocity
M	1	Nov 17	195	179.5	112.5	98.2	7
M	2	Nov 19	205	177.8	107.4	92.9	5
M	3	Nov 20	227	182.3	99.0	76.1	6
M	4	Nov 23	183	175.9	109.5	100.0	7
M	5	Nov 23	256	180.6	123.4	113.7	6
M	6	Nov 24	202	180.1	104.6	90.5	6
M	7	Nov 24	224	178.2	88.8	63.2	8
M	8	Nov 26	210	199.5	104.4	81.3	6
M	9	Nov 29	190	164.7	81.8	61.3	8
M	10	Dec 1	213	190.1	105.1	88.1	6
R	1	Nov 17	186	181.4	108.3	94.3	7
R	2	Nov 19	195	163.6	80.3	56.2	8
R	3	Nov 20	222	174.4	91.6	66.7	8
R	4	Nov 23	199	180.6	110.9	98.9	7
R	5	Nov 23	198	168.3	91.8	72.3	7
R	6	Nov 24	197	180.0	100.0	85.1	7
R	7	Nov 24	216	172.5	90.3	71.0	8
R	8	Nov 26	213	187.2	101.1	84.8	6
R	9	Nov 29	221	173.0	84.8	62.4	8
R	10	Dec 1	197	185.1	106.0	91.4	6

VCL, curvilinear velocity; VAP, velocity of the average path; VSL, straight line velocity.

Sperm were examined with an inverted phase-contrast Leica microscope using a 20× objective. A mounted Prosilica GE680 monochrome camera (Allied Vision Technologies, Burnaby, British Columbia, Canada) recorded images at 200 frames/sec directly to a computer. Sperm that were swimming just above the surface of the slide were analyzed. No cover slip was used. Video was analyzed from 10 to 41 sec post-activation, after which time nearly all sperm in all treatments had stopped moving (see Results).

**Figure 2:**
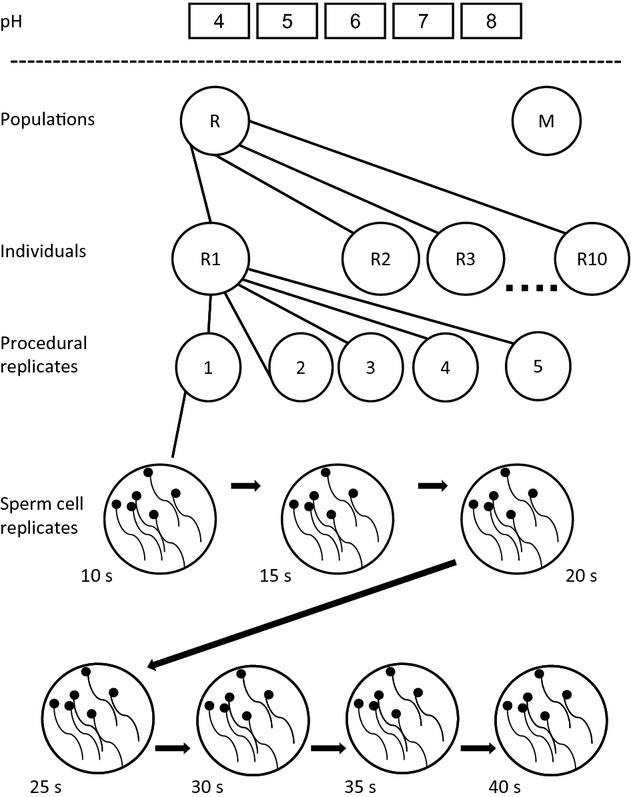
Study design schematic showing hierarchical setup in measures of swimming velocities of individual sperm cells; see text.

Sperm were assessed using water of five different pH (4, 5, 6, 7, 8) levels obtained by adding H_2_SO_4_ or B_4_Na_2_O_7_ to hatchery-sourced freshwater that included 0.1% BSA to prevent sperm from sticking to the slides (Dziewulska et al. 2011). This wide pH range was chosen to push the limits of known reproduction and to introduce environmental stress. New test water was made each day, and pH was measured before and after sperm assessment to ensure stability. Sperm swimming ability was quantified at seven time periods post-activation (10, 15, 20, 25, 30, 35, 40 sec = repeated measures), by analyzing 100 frames of video sequence (0.5 sec) using a more efficient form (Purchase and Earle, in press) of the ImageJ computer assisted sperm analysis (CASA) plugin developed by Wilson-Leedy and Ingermann (2007). The input parameters used for the plugin are available (see Table S1). The entire process was repeated five times, giving 3500 groups of sperm for analysis (2 pops × 10 fish × 5 pH × 7 times × 5 replications); with typically 100–300 total sperm in each group (Fig. 2). A total of 68,496,600 sperm images were used as data after the filters from Table S1 were applied.

The sperm trait most tightly linked to fertilization in brown trout is not known. Of the parameters derived from CASA (Wilson-Leedy and Ingermann 2007), we chose to use curvilinear velocity (VCL) as a measure of sperm quality because it has been shown to be a good predictor of fertilization success in the closely related Atlantic salmon (Gage et al. 2004), and in our experiment, it was correlated with the other measures of velocity (*N* = 261,881 moving sperm; VCL-VAP *r* = 0.82, VAP-VSL *r* = 0.92, VCL-VSL *r* = 0.67).

### Controls

To ensure that any effect of our manipulated water chemistry was due entirely to pH, we setup a control whereby sperm swimming performance was assessed under ambient water conditions (pH 6.6 with the addition of the BSA), and with water that had been first lowered to pH 4.0, then raised to pH 8.0, and then lowered back to pH 6.6. Given identical pH, there should be no difference in sperm performance in these two water sources. This was determined using sperm from four fish (each procedurally replicated five times, as above; see Fig. S1). The controls exhibited identical sperm swimming velocities in adjusted and unadjusted water of the same pH (see Fig. S1), indicating that the chemical additions did not have any confounding effects.

### Statistics

To determine the effects of pH on VCL (the response variable of interest), sperm cells within a procedural replicate were averaged, and then the five procedural replicates were averaged to get data for statistical analysis at the individual fish level. Mean VCL includes only sperm that are motile (sperm with a VCL of 0 are not included in the mean). The independent variables were population (random), individual (random) nested within population, pH (fixed, repeated measures), time post-activation (fixed, repeated measures; Table 2). These were analyzed in a mixed-model nested repeated-measures balanced analysis of variance (ANOVA). Required interaction terms were also included and were specifically assigned to obtain correct error terms for repeated-measures (within-subjects) variables (Table 2). Assumptions of parametric statistics were checked by examining model residuals; *α* was set to 0.05. Statistics were run in R and Minitab software packages.

**Table 2:** Mixed-model nested repeated-measures balanced analysis of variance for brown trout sperm performance (mean VCL) to pH. Only times of 10–25-sec post-activation are included in the analysis as longer periods (30–40 sec) had missing data (no motile sperm in some cases), which unbalanced the design

Source*	Term	df	Error	*F* ratio, *P*-value
1	Population	1	2	0.73, 0.405
2	Individual (population)	18	11	84.63, <0.001
3	pH	4	5	59.86, <0.001
4	Population × pH	4	5	0.60, 0.661
5	pH × individual (population)	72	11	6.85, <0.001
6	Time	3	8	1860.81, <0.001
7	Population × time	3	8	0.85, 0.472
8	Time × individual (population)	54	11	2.72, <0.001
9	pH × time	12	11	61.69, <0.001
10	Population × pH × time	12	11	0.83, 0.624
11	Error	216		
	Total	399		

* “Source” is a code for the different terms, “df” is degrees of freedom, “Error” refers to which “Source” is used in the denominator of the *F*-test. The term pH × time × individual (population) is not included in the model; this appears as error (Source 11) and is the correct error term for testing five of the other terms. Sperm velocity (VCL) is the mean among procedural replicates of the mean among sperm cells within a procedural replicate. The model explained 98% of the variance.

## Results

**Figure 3:**
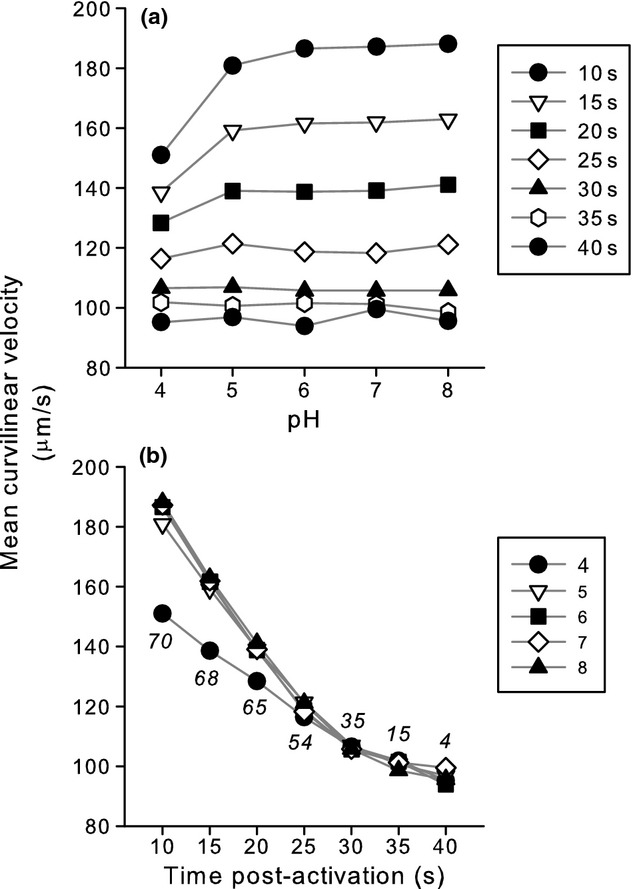
Species-level average reaction norms of brown trout sperm exposed to different environmental pH levels. The two panels are the same data plotted in different ways to aid in interpretation; each line is a (a) time post-activation, (b) pH treatment. Each datum is the mean among 20 individuals, of the mean among five replicates (= 100 microscope procedures), of the mean among moving sperm cells on a slide over a period of 0.5 s. Error bars are not shown; individual data area presented in Figure 5. Numbers inserted on panel (b) are the average percent of sperm that are moving after a given time post-activation (across all procedures, fish, and pH levels).

Brown trout sperm swimming velocity showed plasticity to water pH (Table 2, Fig. 3a), but the reaction norm was neither linear nor consistent. The species-level average reaction norm at 10-sec post-activation was canalized from pH 8–5, but dropped rapidly at pH 4. pH had no influence on sperm cells that had been swimming for more than 20 sec (Fig. 3a), indicating plasticity in plasticity (pH × Time interaction, Table 2). The fastest 10% of moving cells (Serrano et al. 2006) responded similarly to pH as the mean of all moving cells, and are thus not interpreted further. Sperm swimming velocities slowed dramatically with time, but less so at pH 4, as these were moving slowly from the onset (Fig. 3b). Virtually, no motion was present after 40 sec from contact with water (few motile sperm account for the velocity data at 30–40-sec post-activation, Fig. 3b).

**Figure 4:**
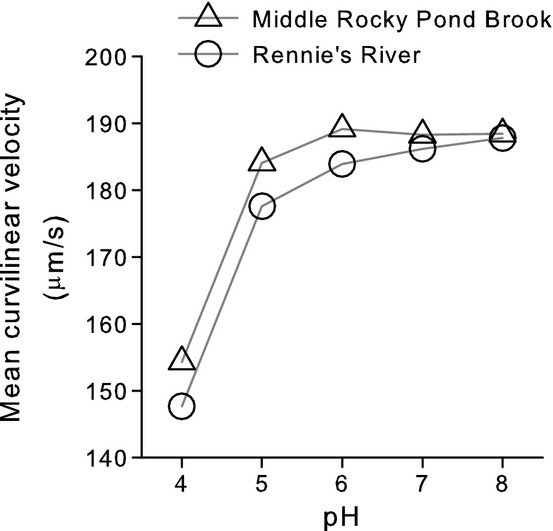
Population-level average reaction norms of brown trout sperm exposed to different environmental pH levels at 10 sec post-activation. Each datum is the mean among 10 individuals, of the mean among five replicates (= 50 microscope procedures), of the mean among moving sperm cells on a slide. Error bars are not shown; individual data are presented in Figure 5.

**Figure 5:**
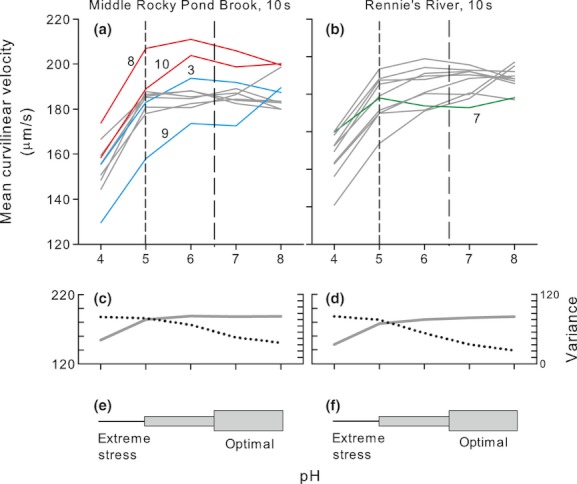
Individual reaction norms of brown trout sperm performance when exposed to different environmental pH levels at 10 sec post-activation. Each line in the top panels (a, b) is the response of sperm from an individual fish and is created from five pH data points (symbols removed), each of which is the mean of five procedural replicates. Middle panels (c, d) follow inserts in figure 2 of Nussey, Wilson, and Brommer (2007) and are the population average (solid line) and variance (dotted line) across environments; on the same *x*-axis scale as the main figures and a *y*-axis scale of 120–220 for the mean (left axis) and 0–120 for variance (right axis). Degree of reproductive stress is indicated on the bottom panels (e, f) following a similar approach as figure 3 in Ghalambor, McKay, Carroll, and Reznick (2007). Breaks in the top panels are transitions from optimal to stressful (loose dashed line), and stressful to extremely stressful (tight dashed line) environments (see Introduction). Numbers (and colours) inserted on the top panels are referred to in the text and match those in Table 1.

Stressful environments revealed cryptic variation. Population-level average reaction norms were similar for these streams (Table 2, Fig. 4). However, reaction norms varied among individuals (an Individual × Environment interaction, Table 2, Fig. 5). Six individuals had peak swimming velocities at pH 8, six at pH 7, seven at pH 6, and one at pH 5 (Table 1). All individuals performed relatively poorly at pH 4, but whether the reaction norm rises or falls between pH 5–6, 6–7, or 7–8 was variable (Fig. 5). Some individuals expressed a relatively canalized phenotype across pH 5–8 (e.g., Rennie's River #7 green line in Fig. 5), whereas others had a domed shaped (e.g., #3 blue, #8 red line), or nearly linear (e.g., #9 blue line) reaction norm. Variance among individuals increased greatly with decreasing pH, a pattern that was similar for both populations (Fig. 5 middle panels). In other words, environments that would be most stressful for reproduction expressed the most variable phenotypes among individuals. For example, individuals labeled #8 and #10 (red lines) have similar phenotypes under optimal pH 8, but are very divergent below pH 6. The pattern is even more pronounced for individuals #3 and #9 (blue lines).

## Discussion

Reaction norms of the plastic expression of phenotypes can vary by genotype, but detecting this can be very difficult. Furthermore, whether such responses are adaptive depends on the trait and context, and interpretation cannot be extrapolated to environments beyond those tested. We documented phenotypic plasticity in brown trout sperm performance and found the average reaction norm within and between populations to be flat over a broad range in river pH. Without finer resolution for individual fish, we would interpret the canalization of the species- and population-level reaction norms as being adaptive. This conclusion is altered when we examine reaction norms of specific individuals, a level at which the conceptual underpinnings are based, but where data are usually lacking (Nussey et al. 2007; Purchase et al. 2010). Stressful environments exposed cryptic variation at the individual level.

Faster swimming sperm are superior at fertilizing eggs. This has been shown experimentally in the sister species of brown trout, Atlantic salmon (Gage et al. 2004). Importantly, it is swimming speed and not sperm number, percent motile, or duration of motility that most directly influences fertilization. Indeed, some have argued that within an ejaculate, it is only the fastest of the sperm that are likely to achieve success (Serrano et al. 2006). Species such as salmon and trout have evolved under sperm competition, and thus it is important to be the first sperm to enter the egg micropyle. To achieve this, timing of gamete release is important as even a 2-sec delay between males significantly reduces fertilization success (Yeates et al. 2007). Sperm that swim slower may swim for longer periods, but this is likely to be maladaptive as faster swimmers will enter the micropyle first. Moreover, moving river water will wash sperm away from the egg within seconds. Although it may be costly for males to produce or maintain high quality semen (see examples in Rudolfsen et al. 2006; Serrano et al. 2006; Gasparini et al. 2010), there is no conceivable benefit of sperm from a given ejaculate to not swim at maximum speeds. Individuals that are likely to experience high sperm competition therefore need to make high-quality semen. For instance, in Atlantic salmon, anadromous males may or may not experience sperm competition during spawning, whereas precocial males typically do. As expected, mature parr invest relatively more into ejaculates and win in sperm competition trials with anadromous males (Vladic and Jarvi 2001).

Superior sperm performance is therefore a key component to male reproductive success. This is tailored internally by semen quality, but in external fertilizers, sperm fertilization capacity is also likely influenced by the hostile and highly variable environment into which sperm are released. Moreover, single-celled sperm are probably more sensitive to environmental variability than the fish themselves. Due to local geology and physical separation among watersheds, freshwater chemistry may vary tremendously within the same climatic zone of a species' range. Anadromous fish such as brown trout spawn in freshwater, but juveniles, may migrate to the ocean to feed. Although most likely return to their natal rivers to spawn, some may stray and ascend a different watershed. Hence, it is critical that trout be able to reproduce in foreign systems that may vary greatly from the environment of their parents. Given its significance in fertilization, it is important that optimal sperm swimming velocities be maintained in different environments. Accordingly, without additional information, we would conclude that the flat average reaction norms over a wide pH range observed here to be adaptive. Moreover, this would also help explain why brown trout are successful colonizers when transplanted from their native range, as it would allow males to do their part in reproduction in a wide range of river systems.

In this study, reaction norms for individuals were highly variable. Although the average reaction norm between and within populations is canalized over a wide pH range, it is obvious that this masks interpretation of inconsistent plasticity among fish. These results are important in three contexts. First, the relative ranking of sperm quality among males varies with pH. As a result, we would predict that if sperm competition experiments were conducted, the winner would depend upon test pH. Second, the ability to make such a comparison of adaptive consequences of reaction norms at different genotypic levels is rare, as it is difficult to determine trait plasticity for individual animals (Nussey et al. 2007; Purchase et al. 2010). Nussey et al. (2007) argue that reaction norms of individuals may not necessarily reflect that of the specific genotype as the individual's past experiences may impart a non-genetic component to the reaction norm. This would apply to studies of sperm from external fertilizers if the animals were taken from the wild. In our study, the captive bred males were exactly the same age, ate exactly the same food, and were reared under common conditions for their entire lives. Therefore, the individual reaction norms shown should reflect that of the genotype (Purchase et al. 2010) and our I × E interaction can serve as a proxy for a G × E interaction. Finally, this variability increased with decreasing pH, supporting the opinions of Ghalambor et al. (2007) that stressful environments should release cryptic variation among individuals. Moreover, the general mean response of slow sperm swimming velocities under harsh conditions is an example of maladaptive plasticity (Ghalambor et al. 2007).

Extreme environments are very useful for studying reaction norms. Genetic assimilation may be a common way in which evolution proceeds in new environments (Schlichting and Pigliucci 1998; West-Eberhard 2003), but there must be genetic variation in plasticity for this to occur. In this context, our results illustrate the importance of examining phenotypic plasticity in response to novel environments. Our significant I × E (and inferred G × E) interaction was most pronounced at pH 4, an extremely stressful environment for salmonid reproduction, and generally increased with decreasing pH; that is increasing among-individual variability (Nussey et al. 2007), through release of cryptic variation (Ghalambor et al. 2007). Ghalambor et al. (2007) describe this as tension being released at the ends of a reaction norm that is stretched like a tight string. Eshel and Matessi (1998) suggest that canalization breaks down under extreme environments, and thus stress can be used to uncover hidden variation among individuals within a given population. In such situations, most novel phenotypes are likely to be less viable; however, if some deviate in a fitness-positive direction, this would be adaptive. In our experiment, this would result from individuals maintaining a relatively canalized phenotype with reduction in pH. Thus, we would predict that such fish would have a clear reproductive advantage over others if they strayed into a foreign river of low pH. Their superior genotypes in this regard could not be identified under benign conditions at pH 8.

Our study carefully examined how sperm performance is shaped by the water chemistry in which they swim. There is, however, an obvious limitation in that we did not manipulate the chemistry of the water in which the males were living. How acclimating these fish to different environments would have affected the response of their sperm to different environments is not known. Given males can sometimes modify sperm and seminal fluid characteristics, one might predict that if a canalized reaction norm is adaptive, the ability to achieve this may be even more pronounced than demonstrated by the sperm of our fish.

Populations may show different patterns in average plasticity, through adaptation to local environments. Our “species-level” reaction norm should therefore be interpreted with caution as only two populations were used in this study. These were chosen through convenience and not because of substantial environmental or genetic variability between them. It is thus possible that the average reaction norm of the species may change if more populations were incorporated. Our results are consistent with other studies on *Salmo* sperm. The swimming velocities reported here are similar to those obtained for brown trout by Dziewulska et al. (2011). Although they did not measure swimming veloicites, Ciereszko et al. (2010) found brown trout sperm could be motile over a pH range of 5.5–10.5. In salmon Daye and Glebe (1984) reported little change in fertilization rates from pH 6.8 to 5.0, but successful fertilization was not obtained at pH 4.0. A general review of effects of pH on fish sperm is provided by Alavi and Cosson (2005).

In summary, individuals differed in how they expressed an important phenotypic trait in response to environmental change, and among-individual variation increased under stressful conditions. Population averages masked this variability. Maintenance of relatively high sperm swimming velocities over a very large range in water chemistry may help explain why brown trout are able to invade such a wide variety of river systems.

## Supporting Information

**Table S1.** Parameters used to set the ImageJ CASA plugin.

**Figure S1.** Sperm swimming velocities of the controls.

## References

[b1] Alavi SMH, Cosson J (2005). Sperm motility in fishes. I. Effects of temperature and pH: a review. Cell Biol. Int.

[b2] Baccetti B, Afzelius BA (1976). The biology of the sperm cell.

[b3] Ciereszko A, Dietrich GJ, Dietrich MA, Nynca J, Kuzminski H, Dobosz S (2010). Effects of pH on sperm motility in several Salmoniformes species: *Oncorhynchus mykiss*
*Salvelinus fontinalis*
*Salmo trutta*
*Salmo salar* and *Thymallus thymallus*. J. Appl. Ichthyol.

[b4] Davidson AM, Jennions M, Nicotra AB (2011). Do invasive species show higher phenotypic plasticity than native species and, if so, is it adaptive? A meta-analysis. Ecol. Lett.

[b5] Daye PG, Garside ET (1977). Lower lethal levels of pH for embryos and alevins of Atlantic salmon, *Salmo salar* L. Can. J. Zool. Revue Canadienne De Zoologie.

[b6] Daye PG, Glebe BD (1984). Fertilization success and sperm motility of Atlantic salmon (*Salmo salar* L.) in acidified water. Aquaculture.

[b7] DeWitt TJ, Sih A, Wilson DS (1998). Costs and limits of phenotypic plasticity. Trends Ecol. Evol.

[b8] Dziewulska K, Rzemieniecki A, Domagala J (2011). Sperm motility characteristics of wild Atlantic salmon (*Salmo salar* L.) and sea trout (*Salmo trutta m. trutta* L.) as a basis for milt selection. J. Appl. Ichthyol.

[b9] Eshel I, Matessi C (1998). Canalization, genetic assimilation and preadaptation: a quantitative genetic model. Genetics.

[b10] Gage MJG, Macfarlane CP, Yeates S, Ward RG, Searle JB, Parker GA (2004). Spermatozoal traits and sperm competition in Atlantic salmon: relative sperm velocity is the primary determinant of fertilization success. Curr. Biol.

[b11] Gasparini C, Simmons LW, Beveridge M, Evans JP (2010). Sperm swimming velocity predicts competitive fertilization success in the green swordtail Xiphophorus helleri. Plos One.

[b12] Gavrilets S, Scheiner SM (1993). The genetics of phenotypic plasticty. 6. Theoretical predictions for directional selection. J. Evol. Biol.

[b13] Ghalambor CK, McKay JK, Carroll SP, Reznick DN (2007). Adaptive versus non-adaptive phenotypic plasticity and the potential for contemporary adaptation in new environments. Funct. Ecol.

[b14] Hustins D (2007). Brown trout and rainbow trout: a journey into Newfoundland waters.

[b15] Jonsson B, Jonsson N (2011). Ecology of Atlantic salmon and brown trout: habitat as a template for life histories.

[b16] Lowe S, Browne M, Boudjelas S, De Poorter M (2000). 100 of the world's worst invasive alien species. A selection from the global invasive species database.

[b17] Nussey DH, Wilson AJ, Brommer JE (2007). The evolutionary ecology of individual phenotypic plasticity in wild populations. J. Evol. Biol.

[b18] Peterson RH, Daye PG, Metcalfe JL (1980). Inhibition of Atlantic salmon (*Salmo salar*) hatching at low pH. Can. J. Fish. Aquat. Sci.

[b19] Price TD, Qvarnstrom A, Irwin DE (2003). The role of phenotypic plasticity in driving genetic evolution. Proc. R. Soc. Lond. B Biol. Sci.

[b20] Purchase CF, Earle PT Modifications to the ImageJ computer assisted sperm analysis (CASA) plugin greatly improve efficiency and fundamentally alter the scope of attainable data. J. Appl. Ichthyol.

[b21] Purchase CF, Butts IAE, Alonso-Fernandez A, Trippel EA (2010). Thermal reaction norms in sperm performance of Atlantic cod (*Gadus morhua*. Can. J. Fish. Aquat. Sci.

[b22] Richards CL, Bossdorf O, Muth NZ, Gurevitch J, Pigliucci M (2006). Jack of all trades, master of some? On the role of phenotypic plasticity in plant invasions. Ecol. Lett.

[b23] Rudolfsen G, Figenschou L, Folstad I, Tveiten H, Figenschou M (2006). Rapid adjustments of sperm characteristics in relation to social status. Proc. R. Soc. B Biol. Sci.

[b24] Schlichting CD, Pigliucci M (1998). Phenotypic evolution: a reaction norm perspective.

[b25] Serrano JV, Folstad I, Rudolfsen G, Figenschou L (2006). Do the fastest sperm within an ejaculate swim faster in subordinate than in dominant males of Arctic char?. Can. J. Zool. Revue Canadienne De Zoologie.

[b26] Serrano I, Buffam I, Palm D, Brannas E, Laudon H (2008). Thresholds for survival of brown trout during the spring flood acid pulse in streams high in dissolved organic carbon. Trans. Am. Fish. Soc.

[b27] Vladic TV, Jarvi T (2001). Sperm quality in the alternative reproductive tactics of Atlantic salmon: the importance of the loaded raffle mechanism. Proc. R. Soc. Lond. B Biol. Sci.

[b28] West-Eberhard MJ (2003). Developmental plasticity and evolution.

[b29] Westley PAH (2011). What invasive species reveal about the rate and form of contemporary phenotypic change in nature. Am. Nat.

[b30] Westley PAH, Fleming IA (2011). Landscape factors that shape a slow and persistent aquatic invasion: brown trout in Newfoundland 1883–2010. Divers. Distrib.

[b31] Wilson-Leedy JG, Ingermann RL (2007). Development of a novel CASA system based on open source software for characterization of zebrafish sperm motility parameters. Theriogenology.

[b32] Woltereck R (1909). Weitere experimentelle Untersuchungen über Artveränderung, speziell über das Wesen quantitativer Artunterscheide bei Daphnien. Verh. Dtsch. Zool. Ges.

[b33] Yeates S, Searle J, Ward RG, Gage MJG (2007). A two-second delay confers first-male fertilization precedence within *in vitro* sperm competition experiments in Atlantic salmon. J. Fish Biol.

