# Supplementary material for: Stressful environments induce novel phenotypic variation: hierarchical reaction norms for sperm performance of a pervasive invader
Source: Ecol Evol. 2012 Sep 13;2(10):2567–76. doi: 10.1002/ece3.364 (PMC3492782; doi:10.1002/ece3.364)
Supplement: Supplementary file 1 [file ece30002-2567-SD1.doc]

Table S1: Parameters used to set the ImageJ CASA plugin. Threshold values of upper=0, lower=170 were applied to all images prior to plugin use.

| Plugin parameter | Value used |
| --- | --- |
| Minimum sperm size (pixels) | 2.0 |
| Maximum sperm size (pixels) | 12.0 |
| Minimum track length (frames) | 100.0 |
| Maximum sperm velocity between frames (pixels) | 5.0 |
| Minimum VSL for motile (µm/s) | 3.0 |
| Minimum VAP for motile (µm/s) | 20.0 |
| Minimum VCL for motile (µm/s) | 25.0 |
| Low VAP speed (µm/s) | 5.0 |
| Maximum percentage of path with zero VAP | 1.0 |
| Maximum percentage of path with low VAP | 25.0 |
| Low VAP speed 2 (µm/s) | 25.0 |
| Low VCL speed (µm/s) | 35.0 |
| High WOB (percent VAP/VCL) | 80.0 |
| High LIN (percent VSL/VAP) | 80.0 |
| High WOB two (percent VAP/VCL) | 50.0 |
| High LIN two (percent VSL/VAP) | 60.0 |
| Frame Rate (frames per second) | 200.0 |
| Microns per 1000 pixels | 956.0 |
